# Exploring the Reliability of Self‐Reported Physical Activity in Aging Populations: A Chilean Cohort Approach

**DOI:** 10.1002/alz70860_107758

**Published:** 2025-12-23

**Authors:** Nicole Rogers, PASITO research group

**Affiliations:** ^1^ Clinica Universidad de los Andes, Santiago, Chile, Universidad de Chile, Santiago, Chile, Instituto de Neurocirugía Asenjo, Santiago, Chile

## Abstract

**Background:**

Physical activity (PA) is a well‐established factor that can help mitigate cognitive decline in aging populations. However, the relationship between PA, sex differences, and cognitive performance remains inadequately explored in underrepresented populations, such as in Latin America. This study aims to provide an initial investigation into the relationship between PA levels and cognitive performance in a cohort of 77 older adults from Chile.

**Method:**

Participants (29 males, 48 females) provided data on their weekly PA, categorized into low, moderate, and high‐intensity activities, through the IPAQ survey. Cognitive function was assessed using MOCA. In addition to PA and cognitive performance, a broad range of demographic, medical, and health‐related variables were collected, including information on physical performance, and medical history. Descriptive statistics were performed to summarize PA and cognitive performance, and differences between sexes and cognitive status, were explored.

**Result:**

On average, participants self‐reported 331.23 minutes/week of PA. Males reported more high‐intensity activity (average 30.34 minutes/week) compared to females (average 10.41 minutes/week), while females engaged more in moderate‐intensity activity (average 37.06 minutes/week). Cognitive scores varied, with an average MOCA score of 21.53 (SE = 0.84). In the amnestic MCI group, participants self‐reported an average of 254.58 minutes/week of total PA, 3.75 minutes/week of high‐intensity activity and 119.7 minutes/week of moderate‐intensity activity. No significant differences were found between cognitively impaired and cognitively healthy individuals. These results do not align with the existing literature, suggesting that there may be significant issues with the reliability of self‐reported surveys.

**Conclusion:**

While sex differences in PA intensity were observed, no significant correlation between PA and cognitive performance was found in this cohort. These findings suggest the need for further research into the impact of PA on cognitive health, particularly exploring additional factors that may influence this relationship. Data collected using wearable fitness devices, already available for this cohort, is being prepared for comparison with self‐reported survey data. This comparison will provide an opportunity to uncover potential discrepancies and question the feasibility of relying on self‐reported surveys for accurate PA measurement, particularly in the cognitively impaired population.

